# Brain Network Constancy and Participant Recognition: an Integrated Approach to Big Data and Complex Network Analysis

**DOI:** 10.3389/fpsyg.2020.01003

**Published:** 2020-06-03

**Authors:** Lu Qiu, Wenya Nan

**Affiliations:** ^1^School of Finance and Business, Shanghai Normal University, Shanghai, China; ^2^Department of Finance, East China University of Science and Technology, Shanghai, China; ^3^Department of Psychology, College of Education, Shanghai Normal University, Shanghai, China

**Keywords:** complex network, symbolic transfer entropy (STE), directed minimum spanning tree (DMST), brain network constancy, participant recognition

## Abstract

With the development of big data sharing and data standardization, electroencephalogram (EEG) data are increasingly used in the exploration of human cognitive behavior. Most of the existing studies focus on the changes of human brain network topology (the number of connections, degree distribution, clustering coefficient phantom) in various cognitive behaviors. However, there has been little exploration into the steady state of multi-cognitive behaviors and the recognition of multi-participant brain networks. To solve these two problems, we used EEG data of 99 healthy participants from the PhysioBank to study multi-cognitive behaviors. Specifically, we calculated the symbolic transfer entropy (STE) between 64 electrode sequences of EEG data and constructed the brain networks of various cognitive behaviors of each participant using the directed minimum spanning tree (DMST) algorithm. We then investigated the eigenvalue spectrum of the STE matrix of each individual's cognitive behavior. The results also showed that the spectrum distributions of different cognitive states of the same participant remained relatively stable, but those of the same cognitive state of different participants varied considerably, verifying the relative stability and uniqueness of the human brain network similar to a human's fingerprint. Based on these features, we used the spectral distribution set of 99 participants of various cognitive states as the original data set and developed a spectral distribution set scoring (SDSS) method to identify the brain network participants. It was found that most labels (69.35%) of the test participant with the highest score were identical to the labeled participant. This study provided further evidence for the existence of human brain fingerprints, and furnished a new approach for dynamic identification of brain fingerprints.

## 1. Introduction

The human brain is a complex and dense network and as such, it has been explored with approaches ranging from 3D maps of brain circuitry (Landhuis, 2017), to communication dynamics in brain networks (Avena-Koenigsberger et al., 2018), and brain evolution (Sporns and Betzel, 2016; Thiran et al., 2016). The varied topological features of the brain network [modular structures (Hearne et al., 2017), network patterns (Vidaurre et al., 2017), nodes and edges (Kawagoe et al., 2017), and structural connectivity (Gu et al., 2018)] can be studied quantitatively (Moon et al., 2017) by techniques such as functional magnetic resonance imaging (fMRI) and electroencephalogram (EEG).

The fMRI (Kim et al., 2016; Wang et al., 2016) is an important quantitative tool to reveal regional functions of the brain. Hadley et al. used graph theory to study the change in brain network topology as a function of treatment response in schizophrenia (Hadley et al., 2016). Shi et al. applied independent component analysis to investigate the large-scale brain network connectivity underlying creativity through the task fMRI data (Shi et al., 2018). Gonzalez et al. validated the utility of the maximum entropy model in describing neurophysiological dynamics by measuring the activation rate in a separate resting state fMRI data set (Gonzalez et al., 2016). Emily et al., using the results of fMRI detection with functional connectivity as the classification standard, identified target participants from a large group of participants. Moreover, recognition was robust so that participants could be accurately identified in both the cognitive behavior and the resting state. They demonstrated that each person's brain connection profile is intrinsic and similar to a "fingerprint" that can be used for participant recognition (Huang J. et al., 2015). Takuya et al. constructed a functional connection network using fMRI detection data and defined the information transmission between the resting functional network and the cognitive behavior network as the transmission network. The information transmission characteristic was used to detect the relationship between the resting network and the cognitive network. It was concluded that the relationship between the cognitive behavior network and the static network was very close. In particular, the resting-state functional network provided a large amount of functional information for the cognitive information network (Ito et al., 2017). The above researchers, using the fMRI image processing and analysis technology, were able to detect the topological structure of participants in each cognitive state. However, the fMRI technique, with its high cost, excels mainly in spatial resolution, but is much less satisfactory with regards to time resolution, which is not conducive to studying brain network dynamics in different time periods.

By contrast, EEG is less accurate than fMRI in spatial positioning, but has a high time resolution at the scale of 1/100 s, lending itself particularly well to the time-window study of the brain network, especially to research brain network dynamics (Kluetsch et al., 2014; Yu et al., 2016; Zippo et al., 2018). Researchers often implemented filtering and independent component analysis (ICA) preprocessing on EEG data (Hatz et al., 2015), calculated the correlation between each two EEG signals, and set a threshold to create a brain network. The methods of calculating the correlation among electrode sequences include Pearson correlation coefficient, granger causality test (Farokhzadi et al., 2017), mutual information (Mikkelsen et al., 2017), and transfer entropy (Centeno and Carmichael, 2014). Among these methods, transfer entropy is the most suitable to reflect the non-linear relationship between brain electrodes. By calculating the transfer entropy between pairs of brain electrodes, one can construct the brain network of different time periods and participants by means of the threshold method or the minimum spanning tree (MST) method. Faes et al. applied entropy-based measures to quantify the predictive information in brain sub-systems and the heart system and identified a structured network of sleeping brain-brain and brain-heart interactions (Faes et al., 2014). Huang et al. calculated the transfer entropy between brain electrodes in drowsy and alert driving states. They concluded that the couplings between pairs of forehead, central lobe, and parietal areas were higher at the vigilance level than in the drowsy driving state (Huang C. S. et al., 2015). Qiao et al. constructed a brain network by fglasso and bootstrapped fglasso for both the alcoholic and the control groups. They found that links of electrodes in the frontal region were denser than those for the control group. In addition, more connected edges were detected in the left central and parietal regions of the alcoholic group (Qiao et al., 2019). Su et al. used MST to unveil the differences of brain network efficiency between young smokers and non-smokers and found that the global network efficiency decreased in young smokers (Su et al., 2017).

The above studies on EEG sequences were mainly based on the change of EEG network topology (network state, network connection number). But there is less research dedicated to quantitative grouping comparisons between EEG networks of cognitive behavior of each participant or considering individual differences among participants. In particular, to our knowledge, no studies have employed a combination of STE and SDSS in EEG analysis. In this study, we aim to investigate the EEG sequences of 99 healthy participants to verify the conclusion of Emily's study (Huang J. et al., 2015) by means of symbolic transfer entropy and spectral analysis. We also seek to explore the potential of using STE and SDSS in participant recognition based on fingerprint characteristics of EEG sequences.

## 2. Materials and Methods

###  Ethics Approval

The datasets for this study are publicly available on https://www.physionet.org/physiobank/database/eegmmidb/ and can be used with no further permission^1^ (Goldberger et al., 2000; Schalk et al., 2004). Since the data have been fully de-identified, no IRB approval is required.

###  EEG Data

**Figure 1:**
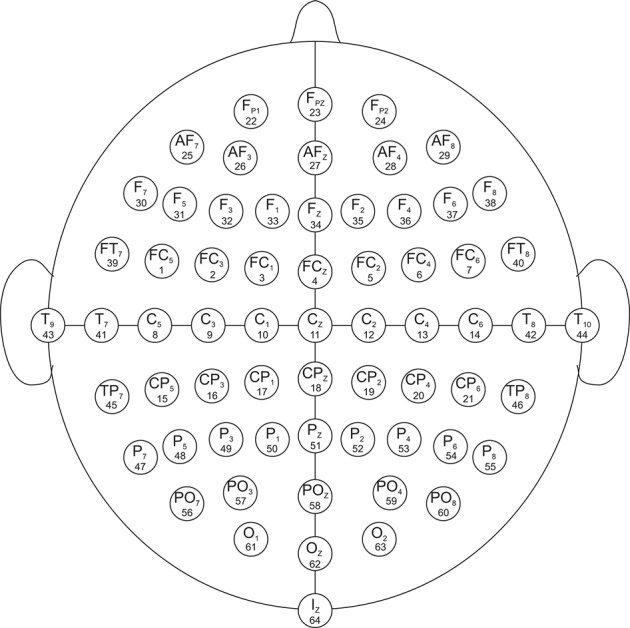
EEG electrode diagram. The EEG are recorded from 64 electrodes which are created by international 10-10 system (excluding electrodes Nz, F9, F10, FT9, FT10, A1, A2, TP9, TP10, P9, and P10). The numbers below each electrode name demonstrate the order in which they appear in the records. The signals in the records are numbered from 1 to 64.

The data set used in this study was created by the developers of the BCI2000 instrumentation system consisting of over 1,500 1- and 2-min EEG recordings, obtained from 99 healthy volunteers. For each participant, voltage values were measured from 64 electrodes as per the international 10-10 system (excluding electrodes Nz, F9, F10, FT9, FT10, A1, A2, TP9, TP10, P9, and P10), shown in Figure 1. All participants were required to perform 14 experimental runs listed in Table 1: two 1-min baseline runs (one with eyes open, one with eyes closed) and three 2-min runs of each of the four following tasks^1^ (Goldberger et al., 2000; Schalk et al., 2004):

1. A target appears on either the left or the right side of the screen. The participant opens and closes the corresponding fist until the target disappears. Then the participant relaxes.
2. A target appears on either the left or the right side of the screen. The participant imagines opening and closing the corresponding fist until the target disappears. Then the participant relaxes.
3. A target appears on either the top or the bottom of the screen. The participant opens and closes either both fists (if the target is on top) or both feet (if the target is on the bottom) until the target disappears. Then the participant relaxes.
4. A target appears on either the top or the bottom of the screen. The participant imagines opening and closing either both fists (if the target is on top) or both feet (if the target is on the bottom) until the target disappears. Then the participant relaxes.

**Table 1:** The 14 experimental runs constructed by different motor/imagery tasks.

**NO**.	**Experimental runs**	**NO**.	**Experimental runs**
1	Baseline, eyes open	8	Task 2
2	Baseline, eyes closed	9	Task 3
3	Task 1 (open and close left or right fist)	10	Task 4
4	Task 2 (imagine opening and closing left or right fist)	11	Task 1
5	Task 3 (open and close both fists or both feet)	12	Task 2
6	Task 4 (imagine opening and closing both fists or both feet)	13	Task 3
7	Task 1	14	Task 4

**Figure 2:**
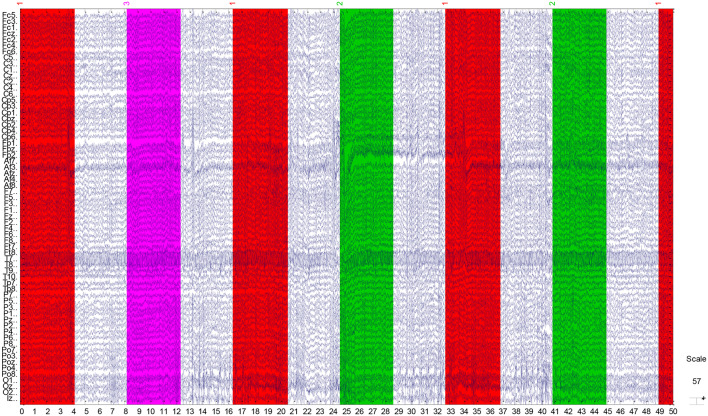
Part signal diagram of task 1 (NO.3 in Table 1) of participant 1. The red, green, and violet regions indicate getting ready for the task, opening the left fist, and opening the right fist, respectively. The white regions mean rest. The horizontal axis shows the elapsed time (second) of the tasks, the vertical axis represents the names of electrodes.

The EEG recordings were input to the EEGLAB toolbox. Each annotation includes one of three codes (e1, e2, or e3): e1 corresponds to rest, e2 corresponds to onset of motion (real or imagined) of the left fist (in runs 3, 4, 7, 8, 11, and 12) and both fists (in runs 5, 6, 9, 10, 13, and 14), and e3 refers to the onset of motion (real or imagined) of the right fist (in runs 3, 4, 7, 8, 11, and 12) and both feet (in runs 5, 6, 9, 10, 13, and 14). The 1-min-runs data of a participant in Task1 are listed in Table 2. Each EEG signal is sampled at 160 points per second. Events in Table 2 include e1, e2, and e3. Latency means the start point of each event. For example, event 1 lasts until points 672, and then event 3 starts at point 1313 (with an intermission of 641 points). The duration means the time span of each event. Part of the corresponding data in Task1 is shown in Figure 2. The red region (event 1) indicates the opening of the eyes when the target appears. The green region (event 2) corresponds to opening the left fist when the target appears on the left. The pink one (event 3) indicates opening the right fist when the target appears on the right. The white one means rest. The horizontal and vertical axes represent the elapsed time (second) and names of electrodes, respectively.

**Table 2:** A participant of event table for task1.

**Number**	**Event**	**Latency**	**Duration**	**Number**	**Event**	**Latency**	**Duration**
**1**	1	1	672	**9**	1	10593	672
**2**	3	1313	656	**10**	3	11905	656
**3**	1	2609	672	**11**	1	13201	672
**4**	2	3921	656	**12**	2	14513	656
**5**	1	5217	672	**13**	1	15809	672
**6**	2	6529	656	**14**	3	17281	656
**7**	1	7825	672	**15**	1	18577	672
**8**	3	9297	656				

###  EEG Signal Pre-Processing and Analysis

We defined the EEG data collection {*G*} as follows:

(1) $$\begin{array}{l} G_{p}^{t}=\left\{g_{p,1}^{t}\left(c,e\right),g_{p,2}^{t}\left(c,e\right),\ldots ,g_{p,N}^{t}\left(c,e\right)\right\} \end{array}$$

where *p* is participant, *t* is task, *c* electrode, *e* event, and *N* the length of sequence. Prior to data analysis, we used eeglab (an interactive matlab toolbox) to filter the EEG sequence and ICA pretreatment. The frequency limit (Kluetsch et al., 2014) was chosen to be 1–70 HZ and 60 Hz notch filtering (Kawagoe et al., 2017). Filter order was automatically chosen (528 recommend) using the function *pop*_*eegfiltnew*()^2^ in eeglab. We used the fully automatic algorithm based on the Independent Components analysis (ICA) algorithm (Mognon et al., 2011) to detect and remove artifacts from the filtered signals. Because the interference signals such as cardiac, eye movement artifacts, and electromyography (EMG) signals are generated by independent sources, ICA decomposition can extract EEG signals from these interference signals. After treatment, the EEG sequence was named {*GQ*},

(2) $$\begin{array}{l} GQ=\left\{gq_{p,1}^{t}\left(c,e\right),gq_{p,2}^{t}\left(c,e\right),\ldots ,gq_{p,N}^{t}\left(c,e\right)\right\} \end{array}$$

The final preprocessing was the first-order difference of the sequence {*GQ*}, and we obtained sequence {*DQ*}:

(3) $$\begin{array}{l} DQ=gq_{p,n+1}^{t}\left(c,e\right)-gq_{p,n}^{t}\left(c,e\right)=dq_{p,n}^{t}\left(c,e\right) \end{array}$$

where *p* = 1, 2…99, *n* = 1, 2, …, *N* − 1, *t* = 1, 2, …, 15(14 experimental runs and 1 rest signal), *c* = 1, 2, …64, *e* = 1, 2, 3(events).

###  Symbolic Transfer Entropy (STE)

After pre-processing, we used transfer entropy to measure the dynamic non-linear relationship of sequences. Transfer entropy is used in many fields, such as the correlation between financial sequences, climate impacts, and EEG/electrocardiogram (ECG) signals. The general formula of transfer entropy is as follows:

(4) $$\begin{array}{ll} TE_{y\to x}^{\left(k,l\right)}=\underset{x_{n+1,}{\text{x}}_{n}^{\left(k\right)},{\text{y}}_{n}^{\left(l\right)}}{\sum }P\left(x_{n+1},x_{n}^{\left(k\right)},y_{n}^{\left(l\right)}\right)\log_{2}\frac{P\left(x_{n+1}|x_{n}^{\left(k\right)},y_{n}^{\left(l\right)}\right)}{P\left(x_{n+1}|x_{n}^{\left(k\right)}\right)} & \; \end{array}$$

where the sequence *X* is a Markov process of degree *k*, and *Y* is a Markov process of degree *l*. The element $x_{n}^{\left(k\right)}$ means that the sequence *X* is influenced by the *k* previous states,and $y_{n}^{\left(l\right)}$ indicates that the sequence *Y* is influenced by the *l* previous states. The parameters *k* and *l* are often set to 1. Then the transfer entropy from variable *Y* to variable *X* is defined as

(5) $$\begin{array}{l} \begin{array}{c} TE_{y\to x}=\underset{x_{n+1,}x_{n},y_{n}}{\sum }P\left(x_{n+1},x_{n},y_{n}\right)\log_{2}\frac{P\left(x_{n+1}|x_{n},y_{n}\right)}{P\left(x_{n+1}|x_{n}\right)}\\ \;=\underset{x_{n+1,}x_{n},y_{n}}{\sum }P\left(x_{n+1},x_{n},y_{n}\right)\log_{2}\frac{P\left(x_{n+1},x_{n},y_{n}\right)P\left(x_{n}\right)}{P\left(x_{n+1},x_{n}\right)P\left(x_{n},y_{n}\right)} \end{array} \end{array}$$

where *P*(*A, B, C*) is the joint probability of *A*, *B*, and *C*, and *P*(*A*|*B*) is the conditional probability of A given by B. Before the calculation of transfer entropy, we translated the sequence {*DQ*} into a symbol sequence. Specifically, we took one sequence from 64 channels for the same participant, same task, and same event as the target research object. For example, in Figure 2, the elapsed times from the 1st second to the 4th second (the horizontal axis) filled in red color means evet1 of task1 (shown in NO.3 of Table 1) of participant 1. We arranged the combined 64 signals in ascending order and divided these data points into three equal parts. The final forms were as follows:

(6) $$\begin{array}{l} \begin{array}{c} B_{p,i}=\left\{\begin{array}{c} 1:T_{p,1}^{t}\left(e\right)\leq dq_{p,n}^{t}\left(c,e\right)<T_{p,\frac{1}{3}L}^{t}\left(e\right)\\ 2:T_{p,\frac{1}{3}L}^{t}\left(e\right)\leq dq_{p,n}^{t}\left(c,e\right)<T_{p,\frac{2}{3}L}^{t}\left(e\right)\\ 3:T_{p,\frac{2}{3}L}^{t}\left(e\right)\leq dq_{p,n}^{t}\left(c,e\right)\leq T_{p,L}^{t}\left(e\right) \end{array}\right\} \end{array} \end{array}$$

where *T* is a new combined sequence of 64 signals. *L* means the length of sequence *T*. *p*, *t*, *c*, and *e* represent participant, task, electrode, and event, respectively. *p* = 1, 2, 3, …, 99, *t* = 1, 2, 3…15, *c* = 1, 2, 3, …64, *e* = 1, 2, 3. We the used phase space reconstruction for symbol EEG signals and set the embedding dimension as 3 (Grassberger and Procaccia, 1983). The correlation between symbol EEG signals was expressed by the (Symbol Transfer Entropy)STE (McAuliffe, 2014).

###  Directed Minimum Spanning Tree (DMST)

By calculating the STE between each two EEG symbol sequences, we obtained the quantitative impact relationships between EEG signals. On this basis, the next vital step was to construct directed brain network diagrams. Using the threshold method to construct directed networks can depict certain brain network structures, but the network constructed by the threshold method is subjective and unstable. In order to ensure the consistency and objectivity of network connections, we made use of the DMST (Gabow et al., 1986; Kwon and Yang, 2008) method to construct the brain network. The minimum spanning tree (MST) algorithm (Crobe et al., 2016) is an important part of graph theory. The classical Kruskal and Prim algorithms of the undirected minimum spanning tree can solve the problem of the symmetrical adjacency matrix. Due to the asymmetry of the transfer entropy matrix, the relations between nodes can be described by DMST, also known as minimum arborescence (Hemminger, 1966). It assigns a special root node to the directed weighted graph. The DMST from the root node requires the minimum total weight of all distance weights. Steps of DMST algorithms are as follows:

1. Select a node as the root node randomly.
2. Travel all edges and find the smallest entry edges of all points except for the root node. Then sum up the weighted values of edges to form the new graph. Determine the final minimum arborescence if no cycles exist in the new graph.
3. If a ring exists in the new graph, shrink the ring into a point and change the edge weight. The way to change edge weights are as follows:
  1. Choose a node *u* in the ring and set the incoming edge of this node as *in*[*u*], and the outgoing edge of this one as (*u, i, w*). *i* and *w* refer to source node and weight, respectively.
  2. Set the new edge weight of node *u* as (*u, i, w* − *in*[*u*]).
  3. Return to Step 2 if the new weight graph contains rings.
4. Expand the new graph if rings do not exist by the breaking loop method (Hemminger, 1966; Gabow et al., 1986). The steps of the breaking loop method were as follows:
  1. Find a loop in the graph.
  2. Remove the edge with the largest weight in the loop, but keep the graph connected.
  3. Repeat this process until there are no loops in the graph (but they are still connected) and get the minimum spanning tree.

###  Average Euclidean Distance and Spectrum Distribution Set Scoring (SDSS)

The brain network constructed using the DMST method can reveal the relation between EEG channels of each participant in each action. The relative stability of events and the difference between participants can be observed in DMST graph. Because of the lack of quantitative analysis in the DMST method, we took the average Euclidean distances as the quantitative parameter indicating the distinctions between brain network patterns:

(7) $$AD_{p}=\frac{\sqrt{\sum_{t=1}^{15}\sum_{e=1}^{3}\sum_{p_{A}=1}^{99}\sum_{p_{B}=1}^{99}\sum_{i=1,j=1}^{64}(TE_{i,j}^{p_{A},e}-TE_{i,j}^{p_{B},e}{)}^{2}}}{15\times 3\times 99\times 99\times 64}$$

(8) $$AD_{e}=\;\frac{\sqrt{\;\sum_{p=1}^{99}\;\sum_{t_{A}=1}^{15}\;\sum_{t_{B}=1}^{15}\;\sum_{e_{A}=1}^{3}\;\sum_{e_{B}=1}^{3}\;\sum_{i=1,j=1}^{64}\;[TE_{i,j}^{t_{A},e_{A}}(p)\;-\;TE_{i,j}^{t_{B},e_{B}}(p){]}^{2}}}{99\times 15\times 15\times 3\times 3\times 64}$$

where *AD*_*p*_ and *AD*_*e*_ indicate the average Euclidean distances of participants and the average Euclidean distances of events, respectively. *e* means an event in each task, *e*_*A*_ and *e*_*B*_ indicate two events in the same task or a different task (*e*_*A*_ = *e*_*B*_ is allowed), *p* means a specific participant out of the 99 participants, *p*_*A*_ and *p*_*B*_ refer to two different participants or the same participant out of the 99 participants (*p*_*A*_ = *p*_*B*_ is also allowed), and *t*_*A*_ and *t*_*B*_ correspond to two tasks from the total 15 tasks.

After quantitative analysis of differences between brain networks, we conducted a union analysis of the brain network by calculating the eigenvalue of the transfer entropy matrix for each participant and event as follows:

(9) $$\begin{array}{l} \lambda_{p}^{t}\left(e,c\right)=\alpha_{p}^{t}\left(e,c\right)+\beta_{p}^{t}\left(e,c\right)∙i \end{array}$$

where α, β indicate real and imaginary parts of the eigenvalues, and *p*, *t*, *e*, and *c* represent participant, task, and event, respectively. *p* = 1, 2…99, *t* = 1, 2…15, *e* = 1, 2, 3, *c* = 1, 2…64. All the eigenvalues were normalized by the Z-Score method and the eigenvalue spectrum distribution of the transfer entropy matrix was shown by the real and imaginary eigenvalues of each action and participant on two-dimensional coordinates. On this basis, we observed and analyzed the spectral distributions of the same events of different participants and different events of the same participants.

**Figure 3:**
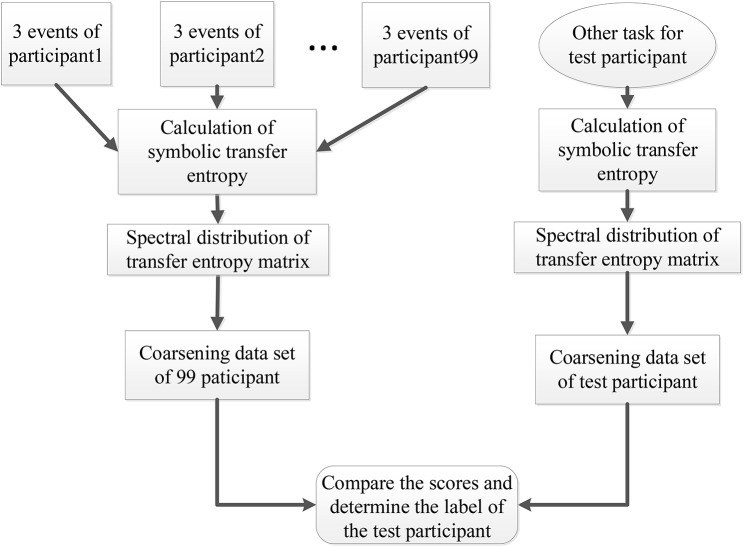
Participant recognition process using the SDSS method. The left part displays the process of constructing the coarsening data set, and the right one indicates the process of calculating the coarsening data set of the test participant. The last step expressed by the rounded rectangle shows the comparison of the data set of 99 participants and the data set of the test participant, to obtain the final score. This final score is used to determine the label of the test participant.

At the same time, the eigenvalues of each action for each participant were conducted to data pre-processing through the coarse graining. First, we took the maximum ($\alpha_{p}^{t}{\left(e\right)}_{\max}$, $\beta_{p}^{t}{\left(e\right)}_{\max}$) and minimum ($\alpha_{p}^{t}{\left(e\right)}_{\min}$, $\beta_{p}^{t}{\left(e,c\right)}_{\min}$) of the real and the imaginary parts of the eigenvalues. Secondly, we defined the scale of coarse-graining θ. Then the ranges of the real part and the imaginary part were defined as $\left\{\alpha_{p}^{t}{\left(e\right)}_{\min}+\theta ,\alpha_{p}^{t}{\left(e\right)}_{\min}+2\theta ,\ldots ,\alpha_{p}^{t}{\left(e\right)}_{\max}-\theta ,\alpha_{p}^{t}{\left(e\right)}_{\max}\right\}$ and $\left\{\beta_{p}^{t}{\left(e\right)}_{\min}+\theta ,\beta_{p}^{t}{\left(e\right)}_{\min}+2\theta ,\ldots ,\beta_{p}^{t}{\left(e\right)}_{\max}-\theta ,\beta_{p}^{t}{\left(e\right)}_{\max}\right\}$ respectively. Finally, we counted the number of actual eigenvalues of different events and participants in this two-dimensional coarsening space. The result was taken as a coarsening data set and used in participant recognition. For participant recognition, the full process of SDSS was shown in Figure 3 with the following steps.

1. We calculated the STE between 64 electrode sequences of each event from the 99 participants and transformed the transfer entropy matrix into the spectral distribution.
2. We created a coarsening data set including the three events (task1) for each of the 99 participants.
3. We selected the data of a participant performing other tasks out of the 99 participants as the test data set and calculated the spectral distribution of the test data set.
4. Finally, we compared the scores and determined the label of the test participant.

**Figure 4:**
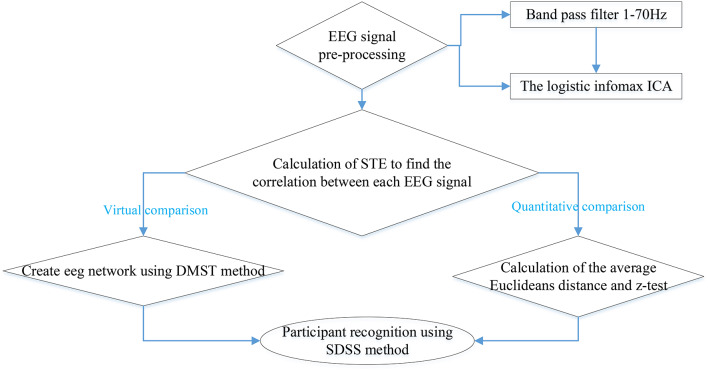
Flowchart of each algorithm.

The entire experiment process is illustrated by a flowchart (Figure 4).

## 3. Results

By means of the above methods, we transformed the EEG signal sequences of the 99 participants into symbolic sequences and calculated the STE of each participant and task. The transfer entropy matrix was transformed into brain networks using the DMST method.

**Figure 5:**
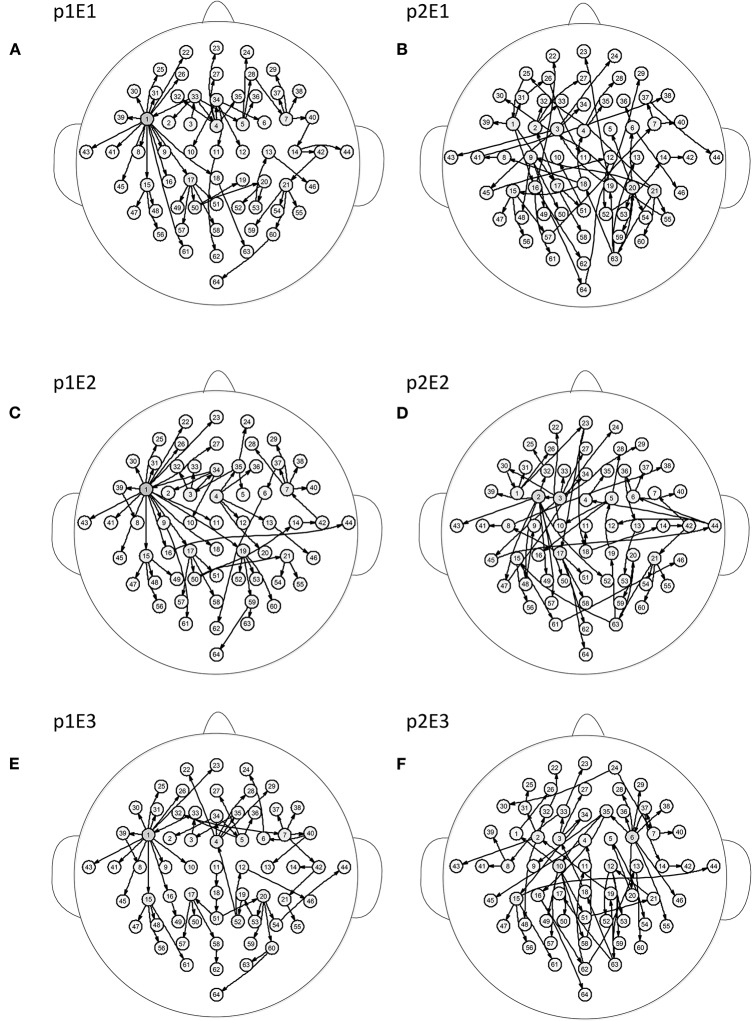
Brain networks of three events of participant 1 and participant 2. The nodes from 1 to 64 correspond to Figure 1. **(A)** event 1 of participant 1 **(B)** event 1 of participant 2 **(C)** event 2 of participant 1 **(D)** event 2 of participant 2 **(E)** event 3 of participant 1 **(F)** event 3 of participant 2.

**Figure 6:**
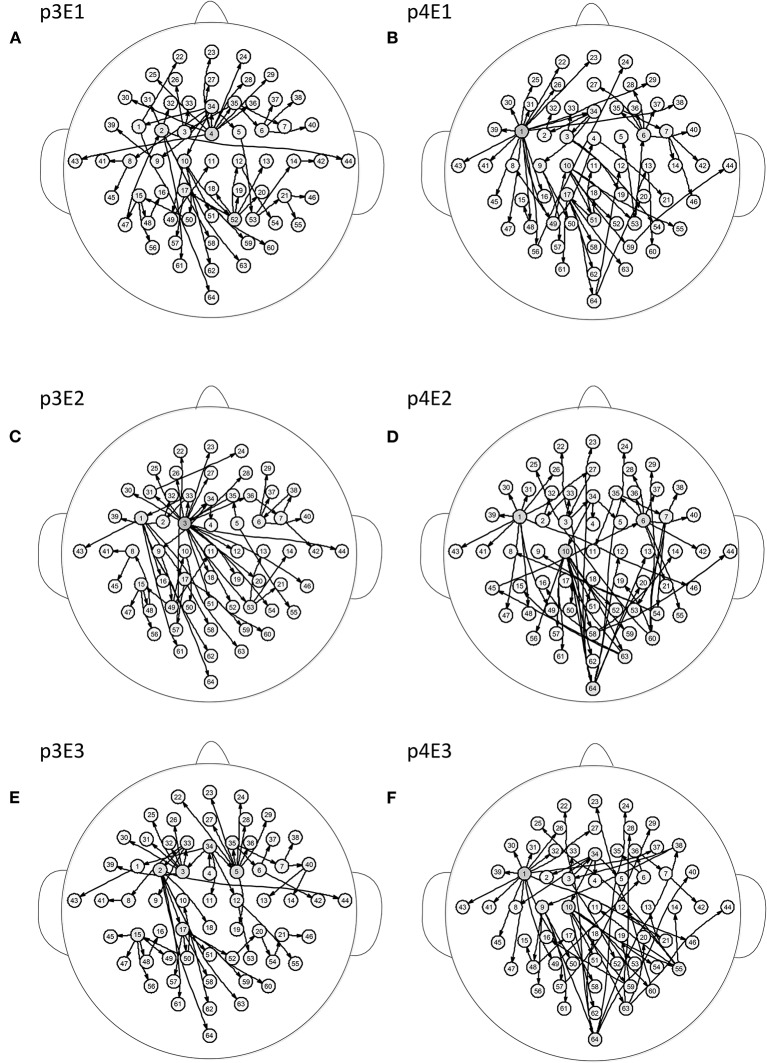
Brain networks of three events of participant 3 and participant 4. The nodes from 1 to 64 correspond to Figure 1. **(A)** event 1 of participant 3 **(B)** event 1 of participant 4 **(C)** event 2 of participant 3 **(D)** event 2 of participant 4 **(E)** event 3 of participant 3 **(F)** event 3 of participant 4.

Figure 5 shows the brain networks of the three events of task 1 for participant 1 and participant 2. For participant 1 in Figure 5A, the node 1(FC5) had the largest out degree which was then treated as the key node in the analysis. In this way, not only can the characteristics of the participants be studied, but the recognition of EEG fingerprints can also be facilitated. At the same time, it can be seen from Figures 5A,C,E that there were little differences among the three brain network graphs of participant 1, which were basically in a constant state. In Figures 5B,D,F, the three brain network diagrams of participant 2 were also basically in a constant state, which showed that the brain network graphs of the same participant in different events had a certain degree of stability. But the same events from different participants, such as p1E1 (event1 of participant 1) and p2E1 (event1 of participant2) in Figures 5A,B, were widely different in structure. Similarly, in Figures 6A,C,E, the network diagrams of the three different events in participant 3 were similar. The three different events in participant 4 also resembled those in Figures 6B,D,F. But the same event of different participants, such as event 1 of participant 3 and participant 4, can be drastically different.

From the results of Figures 5, 6, we can conclude that brain networks of the same participant remain constant to a certain extent regardless of task or rest. The network structures of different participants vary greatly, indicating that everyone has his or her own brain network distribution, similar to a fingerprint, thus lending support to the finding of Emily (Huang J. et al., 2015).

The superposition of brain networks can be used to verify the similarity of networks for different tasks of the same participant, but the error edges arose from the union process lead to information loss in the brain network research. In order to solve this problem, we calculated the eigenvalues of the transfer entropy matrix between EEG recordings of different tasks. The characteristic of the transfer entropy matrix was extracted and then the eigenvalue spectrum was superposed, which not only reveals the basic characteristics of the network, but also achieves the effect of superimposing the common characteristics. Because of the asymmetry of the transfer entropy matrix, the eigenvalues obtained include a real part and an imaginary part. The eigenvalues of different actions between the same participant were extracted and summarized on the coordinate axes.

**Figure 7:**
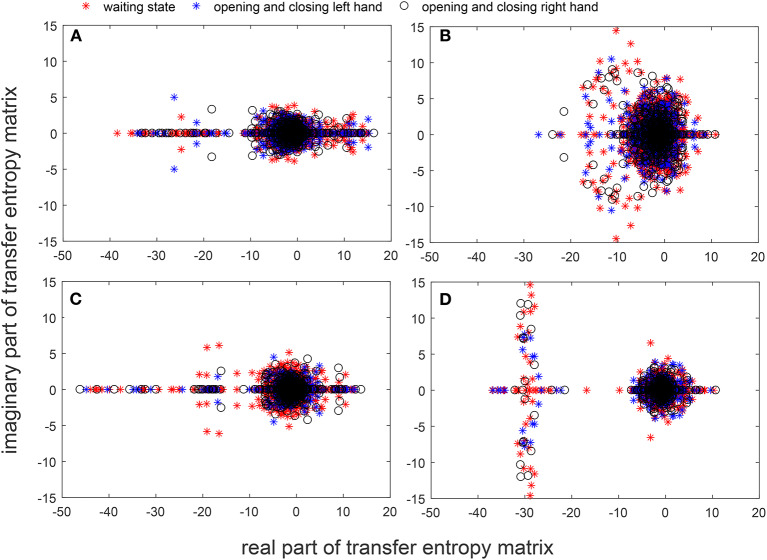
Spectra graphs of transfer entropy matrix. The horizontal axis shows the real part of the transfer entropy matrix, while the vertical axis represents the imaginary part of the transfer entropy matrix. The red star, blue star, and black circle indicate waiting state, opening, and closing the left hand, and opening and closing right hand, respectively. **(A)** 3 events of participant 1 **(B)** 3 events of participant 2 **(C)** 3 events of participant 3 **(D)** 3 events of participant 4.

Figures 7A–D show the spectral distribution of the three actions of participant 1, 2, 3, and 4, respectively. The red star means rest state, the blue star refers to moving the left hand, and the black circle indicates moving the right hand. It can be seen that the spectral structures of the network eigenvalues of the three events of the same participant were very similar, but the spectral structure of each participant obviously differed from each other. The Euclidean distances as quantitative indicators are shown in Tables 3, 4. In Table 3, columns from 2 to 5 indicate the Euclidean distance between the first 4 participants of the same event. The results in column 6 of Table 3 illustrates the mean value of the Euclidean distance of the first 4 participants on the same event. The results in the Table 4 are the Euclidean distances among events of the same participant. Data in Tables 3, 4 are also the corresponding quantitative distances between the left and right networks in Figures 5, 6. From these tables, it can be seen that the average Euclidean distances (36.640, 43.107, 35.767) of participants (from participant 1 to participant 4) in Table 3 were all higher than those (24.792, 25.820, 9.320, 22.154) of events (event1, event2, and event3) in Table 4.

**Table 3:** Euclidean distances among participants of the same event.

**Event 1**	**p1^**a**^**	**p2^**a**^**	**p3^**a**^**	**p4^**a**^**	**AED^**b**^**
p1	0	42.988	36.354	24.143	36.640
p2	42.988	0	46.512	45.141	
p3	36.354	46.512	0	24.704	
p4	24.143	45.141	24.704	0	
	**p1**	**p2**	**p3**	**p4**	**AED**^**b**^
**Event 2**					
p1	0	48.827	51.503	55.224	43.107
p2	48.827	0	42.520	31.579	
p3	51.503	42.520	0	28.989	
p4	55.224	31.579	28.989	0	
**Event 3**					
p1	0	41.039	34.286	36.366	35.767
p2	41.039	0	44.181	36.563	
p3	34.286	44.181	0	22.167	
p4	36.366	36.563	22.167	0	

a *p1, p2, p3, p4, indicate participant1, participant 2, participant 3, participant 4, respectively*.

b *AED, average of Euclidean distances*.

**Table 4:** Euclidean distances among events of the same participant.

**Participant 1**	**Event 1**	**Event 2**	**Event 3**	**AED^**a**^**	**Participant 2**	**Event 1**	**Event 2**	**Event 3**	**AED^**a**^**
Event1	0	20.232	27.630	24.792	event1	0	27.891	18.140	25.820
Event2	20.232	0	26.514		event 2	27.891	0	31.430	
Event3	27.630	26.514	0		event 3	18.140	31.430	0	
**Participant 3**	**Event 1**	**Event 2**	**Event 3**	**AED**^**a**^	**Participant 4**	**Event 1**	**Event 2**	**Event 3**	**AED**^**a**^
Event 1	0	9.954	5.977	9.320	event 1	0	24.310	17.820	22.154
Event 2	9.954	0	12.027		event 2	24.310	0	24.333	
Event 3	5.977	12.027	0		event 3	17.820	24.332	0	

a *AED, average of Euclidean distances*.

In order to statistically analyze the spectral distribution of all participants, we used the two-factor repeated measures ANOVA to test the differences between within-participant and between-participant spectra. Specifically, we transformed the spectrum distribution results into 5760-by-99 matrices (128*3*15 = 5760). The length of each spectrum distribution was 128 including the real part and the virtual part. The numbers of task and event were 15 and 3, respectively. Ninety-nine indicated the participant number. Then we put the matrix into the two-factor repeated measures ANOVA model and obtained the results shown in Table 5.

**Table 5:** The results of the two-factor repeated measures ANOVA.

**Source**	**SS**	**df**	**MS**	**F**	**Prob>F**
Columns	3137.88	98	32.0192	2.17	1.61805*E*−10
Rows	16.24	44	0.3691	0.03	1
Interaction	757.01	4312	0.1756	0.01	1
Error	8330284.5	565785	14.7234		
Total	8334195.5	570239			

In Table 5, the *p*−*value* of the participant factor (between-participant shown by Columns) in the second row was 1.61805 × 10^−10^ < α = 0.01. In the third and fourth rows, the *p* − *value*s of the task factor (within-participant expressed with Rows) and interaction factor equaled 1>α = 0.01. That means between-participant spectrum distributions were significantly different while the within-subject spectrum distributions had no significant difference.

**Figure 8:**
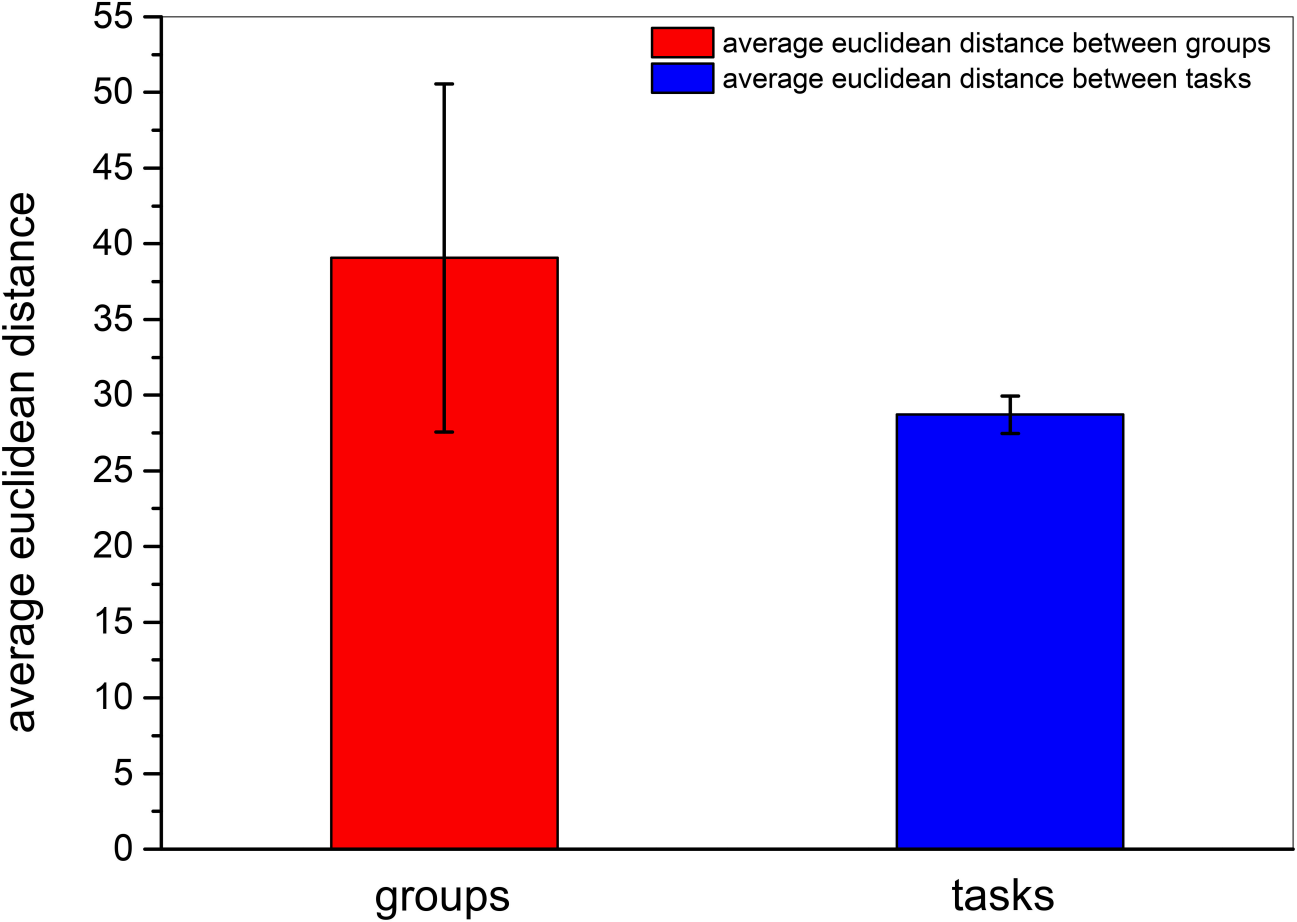
Average Euclidean distance among different participants and among different tasks. The red column and the blue column indicate the average Euclidean distance among participants and the average Euclidean distance among tasks, respectively. The error bars indicate the standard deviations of average Euclidean distances.

We then obtained the quantitative result to confirm that inter-participant differences in the same event were more pronounced than inter-task differences of the same participant. As shown in Figure 8, the quantitative parameter indicating the average Euclidean distance among participants, shown by the red column, was higher than the average Euclidean distance among events represented by the blue column. The standard deviation within the participant group was also higher than that between event groups. In addition, we also compared the Euclidean distance among participants and the Euclidean distance among tasks by *z* − *test*. As presented in Table 6, the average Euclidean distance and standard deviation were the same as shown in Figure 8. The numbers of Euclidean distances were calculated as follows: $\frac{99*98}{2}=4851,\;\frac{\left(15*3\right)*\left(15*3-1\right)}{2}=990$, where 99 was the number of participants, 15 was task number, and each task contained three events. The *z* value was higher than the critical value of both one-tailed and two-tailed tests. The *p* − *value* of the *z* − *test* equaled 0. The results of the *z* − *test* quantitatively demonstrated that the differences between brain networks of participants were larger than the differences between tasks.

**Table 6:** *z* − *test* analysis for two groups of Euclidean distances.

	**Participants**	**Tasks**
Average	39.471	29.362
Standard deviation	11.496	1.546
Numbers of Euclidean distances	4851	990
z	59.564	
P(Z < =z) one-tailed (α = 0.01)	0	
z critical value of one-tailed	2.326	
P(Z < =z) two-tailed (α = 0.01)	0	
z critical value of two-tailed	2.576	

**Figure 9:**
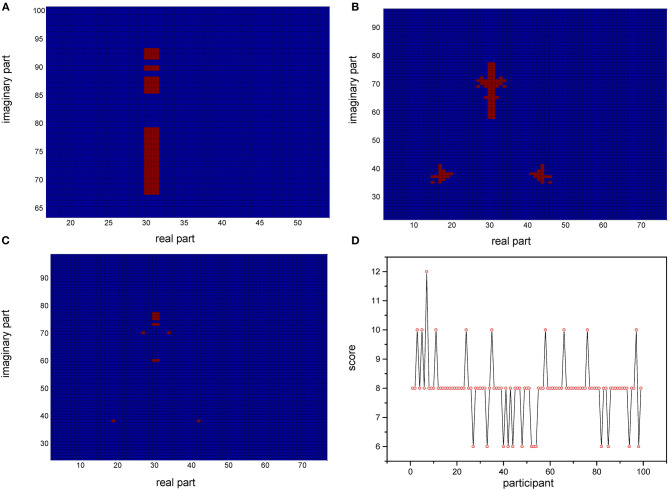
Coarsened spectrum distributions and test scores. **(A)** Coarsened spectrum distribution of task 1 of participant 1. **(B)** Coarsened spectrum distribution of task 1 of participant 7. **(C)** Coarsened spectrum distribution of task 3 of test participant. **(D)** Score of *TXE*3 (task3 of test participant); the horizontal axis shows the participant number; the vertical axis indicates the score (overlapping part of the spectrum of *TXE*3 and data sets).

**Figure 10:**
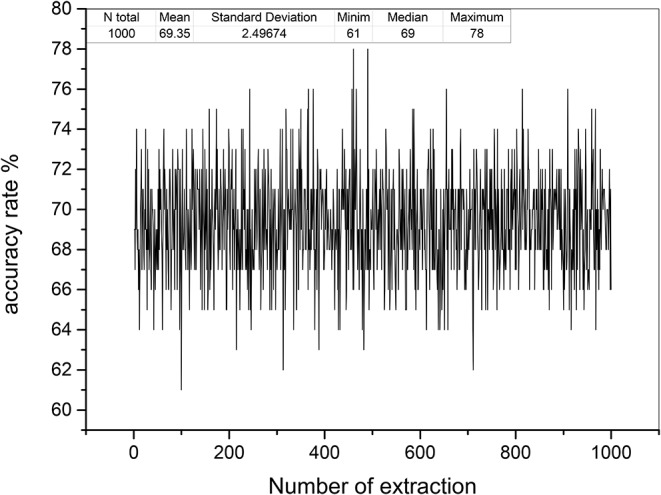
Test accuracy rates of 1,000 test groups. The horizontal axis shows the number of extractions; the vertical axis indicates the accuracy rate (99 was divided by overlapping capacities of the spectrum of each test group and data sets).

Based on the relative stability of brain network of each participant, we used the SDSS method to create data sets using the network spectrum data of three events of 99 participants. When judging the test participants, any task of the test participants, such as moving both legs, can be used as measurement data. We compared the network spectrum structure of the measured participant with 99 participants' data set by coarsening the network spectrum. The choice of the accuracy of coarsening determines the accuracy of the final results. In this paper, we set θ = 1 to divide the spectrograms into various small squares and counted the number of particles in each small square. Finally, a participant test was carried out, assuming that the moving legs of participant 7 in task 3 were selected as measurement actions, labeled as *TXE*3. We calculated the transfer entropy matrix of this labeled task, whose spectrum distribution was coarsened by θ = 1. By comparing the *TXE*3 coarsening data with 99 participants' coarsening data, the number of *TXE*3 was found with the highest score. Figures 9A,B show the spectrum distribution sets of participant 1 and participant 7, respectively. Figure 9C is the spectrum distribution set of *TXE*3. Figure 9D is the test score of *TXE*3. The horizontal axis represents the participant number, and the vertical axis score represents the overlapping part between the spectrum of *TXE*3 and data sets created by the three events of 99 participants. It can be seen that the highest score corresponds to participant 7. That is to say, the test participant was participant 7. This was consistent with the participant number selected beforehand. We also checked all participants of *T*_*new*1 (open and close both fists), *T*_*new*2 (open and close both feet), and *T*_*new*3 (imagine opening and closing both fists) by creating three new groups named *T*_*new*1_*g*, *T*_*new*2_*g*, and *T*_*new*3_*g*. Each group contained 99 participants of the new tasks (*T*_*new*1, *T*_*new*2 and *T*_*new*3). Thirty-three participants were selected without repetition from *T*_*new*1_*g*, *T*_*new*2_*g* and *T*_*new*3_*g*. A new cross test group was then created. We repeated the extraction 1,000 times and created 1,000 test groups. The 1,000 scores are shown in Figure 10 and the average accuracy of test participants is 69.35%, which helped validate the effectiveness of the SDSS method.

## 4. Discussion

EEG network research is regarded as an effective tool in identifying subject specific characteristics. As a core method for creating a network, the MST method assesses the strongest connection of individual EEG traits. Crobe et al. used MST and the k-core decomposition method to find the existence of a distinctive functional core. Their results confirmed the great impact of EEG analysis on several bioengineering applications (Crobe et al., 2016). Compared to the MST method, the DMST method can express the direction between each two nodes in the created EEG network. We can obtain the source node from the EEG network and find some features from it. Gennaro et al. found that the individual EEG-trait remains stable despite the change of sleep architecture. They proposed that EEG invariances can be related to genetic individual differences rather than sleep-dependent mechanisms (De Gennaro et al., 2005). Thomas et al. confirmed that the EEG signals are robust carriers of unique personality traits and reported that future research must focus on the uniqueness, acceptability, and robustness of EEG signals by various optimization algorithms and advanced technology (Thomas and Vinod, 2017). As mentioned in the above literature (De Gennaro et al., 2005; Huang J. et al., 2015; Thomas and Vinod, 2017): the connections in the human brain network are intrinsic and maintains a stable state, similar to the human “fingerprint.” In our research, we also found these stable individual EEG traits using the graphic method (DMST) and quantitative analysis (*z*-test of Euclidean distance). Specifically, we used the eeglab toolbox in MATLAB to load the 20G EEG sequence data of 99 participants and preprocessed the data. The STE method was then used to calculate the transfer entropy of the three events for the 99 participants, and the DMST method was used to generate the brain networks of various cognitive behaviors for each participant. By visual inspection, brain networks of the same participant were very similar in different events, but there were great differences between different participants in the same event. For quantitative analysis, we used *z* − *test* to compare Euclidean distances of participants and events. The results showed that the Euclidean distances between participants were significantly greater than those between events.

In addition, by focusing on this feature (EEG-trait remains stable), we used the SDSS method to construct the respective micro data sets (fingerprint database) based on the coarsened network spectrum of the rest, the left-hand and right-hand tasks of the 99 participants. For participant recognition, we created three groups of test data named by tasknew1 (open and close both fists), tasknew2 (open and close both feet), and tasknew3 (imagine opening and closing both fists). Each group contained 99 participants. We chose 33 different participants from group1, group2, and group3 randomly and created the new disordered group. We repeated the selection 1,000 times and obtained 1,000 new disordered groups. The average accuracy of test groups was 69.35%, which showed the effectiveness of the SDSS method.

## 5. Limitation

This present study is not without limitations: 1. In this paper, we selected the BCI2000 dataset as the research data, but BCI has a critical hurdle, in that performance varies greatly, especially in motor imagery based BCI. Researchers tried to address the problem of performance variation (Ahn and Jun, 2015) to improve reliability. In future studies, we look forward to improving the reliability and to focus the attention on task-related factors and longitudinal tracking of participants as well as integrative studies of related variables (psychological and physiological). 2. This study was limited in catching the flexible and dynamic characteristics of EEG signals when calculating the STE (McAuliffe, 2014). Further studies with the STE of short EEG sequences (about 10^2^ points) (Zhang et al., 2012; Pan et al., 2014) would be required to avoid excessive reduction of brainwave features. 3. The accuracy of the coarse-grained network spectrograms of the 99 participants was likely to affect the final results, thus, in future work, we will try to select a better parameter not only to increase the accuracy of the coarse-grained network spectrogram but also to enhance the speed of identification.

## 6. Conclusion

In conclusion, the spectral analysis in complex networks can provide a very simple computational model for studying the rules of big data (multiple participants and multi-channel EEG). One can use the characteristics of the complex network spectrum to identify EEG participants. In addition, the SDSS method in this paper had important implications for the detailed comparison of network states.

## Data Availability Statement

The datasets generated for this study are available on request to the corresponding author.

## Ethics Statement

The datasets for this study are publicly available on https://www.physionet.org/physiobank/database/eegmmidb/ and can be used with no further permission. Since the data have been fully de-identified, no IRB approval is required.

## Author Contributions

LQ designed the research and performed the calculations. LQ and WN analyzed the data and wrote the paper. All authors contributed to manuscript revision, read, and approved the submitted version.

### Conflict of Interest

The authors declare that the research was conducted in the absence of any commercial or financial relationships that could be construed as a potential conflict of interest.

## Footnotes

^1^https://www.physionet.org/cgi-bin/atm/ATM

^2^https://www.ccn.ucla.edu/wiki/index.php/Hoffman2:MATLAB:EEGLAB:Jobs.

## References

[B1] Ahn M. Jun S. C. (2015). Performance variation in motor imagery brain-computer interface: a brief review. J. Neurosci. Methods 243, 103–110. 10.1016/j.jneumeth.2015.01.033 25668430

[B2] Avena-Koenigsberger A. Misic B. Sporns O. (2018). Communication dynamics in complex brain networks. Nat. Rev. Neurosci. 19, 17–33. 10.1038/nrn.2017.149 29238085

[B3] Centeno M. Carmichael D. W. (2014). Network connectivity in epilepsy: resting state fMRI and EEG-fMRI contributions. Front. Neurol. 5:93. 10.3389/fneur.2014.00093 25071695 PMC4081640

[B4] Crobe A. Demuru M. Didaci L. Marcialis G. L. Fraschini M. (2016). Minimum spanningtree and k-core decomposition as measure of subject-specific EEG traits. Biomed. Phys. Eng. Express 2:017001 10.1088/2057-1976/2/1/017001

[B5] De Gennaro L. Ferrara M. Vecchio F. Curcio G. Bertini M. (2005). An electroencephalographic fingerprint of human sleep. Neuroimage 26, 114–122. 10.1016/j.neuroimage.2005.01.020 15862211

[B6] Faes L. Nollo G. Jurysta F. Marinazzo D. (2014). Information dynamics of brain-heart physiological networks during sleep. New J. Phys. 16:105005 10.1088/1367-2630/16/10/105005

[B7] Farokhzadi M. Soltanian-Zadeh H. Hossein-Zadeh G. A. (2017). “Nonlinear Granger Causality using ANFIS for identification of causal couplings among EEG/MEG time series,” in 2016 23rd Iranian Conference on Biomedical Engineering and 2016 1st International Iranian Conference on Biomedical Engineering, ICBME 2016 (Tehran), 69–73. 10.1109/ICBME.2016.7890931

[B8] Gabow H. N. Galil Z. Spencer T. Tarjan R. E. (1986). Efficient algorithms for finding minimum spanning trees in undirected and directed graphs. Combinatorica 6, 109–122. 10.1007/BF02579168

[B9] Goldberger A. L. Amaral L. A. Glass L. Hausdorff J. M. Ivanov P. C. Mark R. G.. (2000). PhysioBank, PhysioToolkit, and PhysioNet: components of a new research resource for complex physiologic signals. Circulation 101, 215–220. 10.1161/01.cir.101.23.e215 10851218

[B10] Gonzalez C. C. Billington J. Burke M. R. (2016). The involvement of the fronto-parietal brain network in oculomotor sequence learning using fMRI. Neuropsychologia 87, 1–11. 10.1016/j.neuropsychologia.2016.04.021 27157884

[B11] Grassberger P. Procaccia I. (1983). Measuring the strangeness of strange attractors. Phys. D Nonlinear Phenom. 9, 189–208. 10.1016/0167-2789(83)90298-1

[B12] Gu S. Cieslak M. Baird B. Muldoon S. F. Grafton S. T. Pasqualetti F.. (2018). The energy landscape of neurophysiological activity implicit in brain network structure. Sci. Rep. 8:2507. 10.1038/s41598-018-20123-8 29410486 PMC5802783

[B13] Hadley J. A. Kraguljac N. V. White D. M. Ver Hoef L. Tabora J. Lahti A. C. (2016). Change in brain network topology as a function of treatment response in schizophrenia: a longitudinal resting-state fMRI study using graph theory. npj Schizophr. 2:16014. 10.1038/npjschz.2016.14 27336056 PMC4898893

[B14] Hatz F. Hardmeier M. Bousleiman H. Regg S. Schindler C. Fuhr P. (2015). Reliability of fully automated versus visually controlled pre- and post-processing of resting-state EEG. Clin. Neurophysiol. 126, 268–274. 10.1016/j.clinph.2014.05.014 24996926

[B15] Hearne L. J. Cocchi L. Zalesky A. Mattingley J. B. (2017). Reconfiguration of brain network architectures between resting-state and complexity-dependent cognitive reasoning. J. Neurosci. 37, 8399–8411. 10.1523/jneurosci.0485-17.2017 28760864 PMC6596866

[B16] Hemminger R. L. (1966). On the group of a directed graph. Can. J. Math. 18, 210–220. 10.4153/cjm-1966-023-2

[B17] Huang C. S. Pal N. R. Chuang C. H. Lin C. T. (2015). Identifying changes in EEG information transfer during drowsy driving by transfer entropy. Front. Hum. Neurosci. 9:570. 10.3389/fnhum.2015.00570 26557069 PMC4615826

[B18] Huang J. Finn E. S. Chun M. M. Scheinost D. Shen X. Constable R. T.. (2015). Functional connectome fingerprinting: identifying individuals using patterns of brain connectivity. Nat. Neurosci. 18, 1664–1671. 10.1038/nn.4135 26457551 PMC5008686

[B19] Ito T. Kulkarni K. R. Schultz D. H. Mill R. D. Chen R. H. Solomyak L. I.. (2017). Cognitive task information is transferred between brain regions via resting-state network topology. Nat. Commun. 8:1027. 10.1038/s41467-017-01000-w 29044112 PMC5715061

[B20] Kawagoe T. Onoda K. Yamaguchi S. (2017). Associations among executive function, cardiorespiratory fitness, and brain network properties in older adults. Sci. Rep. 7:40107. 10.1038/srep40107 28054664 PMC5215211

[B21] Kim S. Y. Qi T. Feng X. Ding G. Liu L. Cao F. (2016). How does language distance between L1 and L2 affect the L2 brain network? An fMRI study of Korean-Chinese-English trilinguals. Neuroimage 129,25–39. 10.1016/j.neuroimage.2015.11.068 26673115

[B22] Kluetsch R. C. Ros T. Théberge J. Frewen P. A. Calhoun V. D. Schmahl C.. (2014). Plastic modulation of PTSD resting-state networks and subjective wellbeing by EEG neurofeedback. Acta Psychiatr. Scand. 130, 123–136. 10.1111/acps.12229 24266644 PMC4442612

[B23] Kwon O. Yang J. S. (2008). Information flow between stock indices. EPL 82:68003 10.1209/0295-5075/82/68003

[B24] Landhuis E. (2017). Neuroscience: big brain, big data. Nature 541, 559–561. 10.1038/541559a 28128250

[B25] McAuliffe J. (2014). The new math of EEG: Symbolic transfer entropy, the effects of dimension. Clin. Neurophysiol. 125:17 10.1016/j.clinph.2013.12.017

[B26] Mikkelsen K. B. Kidmose P. Hansen L. K. (2017). On the Keyhole hypothesis: high mutual information between ear and scalp EEG. Front. Hum. Neurosci. 11:341. 10.3389/fnhum.2017.00341 28713253 PMC5492868

[B27] Mognon A. Jovicich J. Bruzzone L. Buiatti M. (2011). ADJUST: an automatic EEG artifact detector based on the joint use of spatial and temporal features. Psychophysiology 48, 229–240. 10.1111/j.1469-8986.2010.01061.x 20636297

[B28] Moon J. Y. Kim J. Ko T. W. Kim M. Iturria-Medina Y. Choi J. H.. (2017). Structure shapes dynamics and directionality in diverse brain networks: mathematical principles and empirical confirmation in three species. Sci. Rep. 7:46606. 10.1038/srep46606 28425500 PMC5397857

[B29] Pan X. Hou L. Stephen M. Yang H. Zhu C. (2014). Evaluation of scaling invariance embedded in short time series. PLoS ONE 9:e116128. 10.1371/journal.pone.0116128 25549356 PMC4280174

[B30] Qiao X. Guo S. James G. M. (2019). Functional graphical models. J. Am. Stat. Assoc. 114, 211–222. 10.1080/01621459.2017.1390466

[B31] Schalk G. McFarland D.J. Hinterberger T. Birbaumer N. Wolpaw J.R. (2004). BCI2000: a general-purpose brain-computer interface (BCI) system. IEEE Trans. Biomed. Eng. 51, 1034–1043. 10.1109/TBME.2004.827072 15188875

[B32] Shi L. Sun J. Xia Y. Ren Z. Chen Q. Wei D.. (2018). Large-scale brain network connectivity underlying creativity in resting-state and task fMRI: cooperation between default network and frontal-parietal network. Biol. Psychol. 135, 102–111. 10.1016/j.biopsycho.2018.03.005 29548807

[B33] Sporns O. Betzel R. F. (2016). Modular brain networks. Annu. Rev. Psychol. 67, 613–640. 10.1146/annurev-psych-122414-033634 26393868 PMC4782188

[B34] Su S. Yu D. Cheng J. Chen Y. Zhang X. Guan Y.. (2017). Decreased global network efficiency in young male smoker: an EEG study during the resting state. Front. Psychol. 8:1605. 10.3389/fpsyg.2017.01605 28951727 PMC5599785

[B35] Thiran J.-P. Fischi-Gomez E. Eixarch E. Batalle D. Hüppi P. S. Gratacós E.. (2016). Structural brain network reorganization and social cognition related to adverse perinatal condition from infancy to early adolescence. Front. Neurosci. 10:560. 10.3389/fnins.2016.00560 28008304 PMC5143343

[B36] Thomas K. P. Vinod A. P. (2017). Toward EEG-based biometric systems: the great potential of brain-wave-based biometrics. IEEE Syst. Man Cybern. Mag. 3, 6–15. 10.1109/msmc.2017.2703651

[B37] Vidaurre D. Smith S. M. Woolrich M. W. (2017). Brain network dynamics are hierarchically organized in time. Proc. Natl. Acad. Sci. U.S.A. 114, 12827–12832. 10.1073/pnas.1705120114 29087305 PMC5715736

[B38] Wang L. Wu L. Lin X. Zhang Y. Zhou H. Du X.. (2016). Altered brain functional networks in people with Internet gaming disorder: evidence from resting-state fMRI. Psychiatry Res. Neuroimaging 254, 156–163. 10.1016/j.pscychresns.2016.07.001 27447451

[B39] Yu Q. Wu L. Bridwell D. A. Erhardt E. B. Du Y. He H.. (2016). Building an EEG-fMRI multi-modal brain graph: a concurrent EEG-fMRI study. Front. Hum. Neurosci. 10:476. 10.3389/fnhum.2016.00476 27733821 PMC5039193

[B40] Zhang W. Qiu L. Xiao Q. Yang H. Zhang Q. Wang J. (2012). Evaluation of scale invariance in physiological signals by means of balanced estimation of diffusion entropy. Phys. Rev. E Stat. Nonlinear Soft Matter Phys. 86:056107. 10.1103/PhysRevE.86.056107 23214843

[B41] Zippo A. G. Della Rosa P. A. Castiglioni I. Biella G. E. M. (2018). Alternating dynamics of segregation and integration in human EEG functional networks during working-memory task. Neuroscience 371, 191–206. 10.1016/j.neuroscience.2017.12.004 29246785

